# Meal Duration and Obesity-Related Indicators among Adolescents: Insights from the EHDLA Study

**DOI:** 10.3390/nu16162769

**Published:** 2024-08-20

**Authors:** Mayra Fernanda Martínez-López, José Francisco López-Gil

**Affiliations:** 1 Cancer Research Group, Faculty of Medicine, Universidad de Las Américas, Quito 170124, Ecuador; 2 One Health Research Group, Universidad de Las Américas, Quito 170124, Ecuador; josefranciscolopezgil@gmail.com; 3 Department of Communication and Education, Universidad Loyola Andalucía, 41704 Seville, Spain

**Keywords:** overweight, fatness, body fat, youths, mealtimes, eating speed, mindless eating

## Abstract

Purpose: This paper aims to examine the association between meal duration and obesity indicators among Spanish adolescents. Methods: We conducted a cross-sectional analysis using data from the Eating Healthy and Daily Life Activities (EHDLA) project involving 755 adolescents aged 12 to 17 years (54.8% girls) from three secondary schools in the *Valle de Ricote* Region of Murcia, Spain. To evaluate overall meal duration, participants were asked how long (on average) breakfast, morning snacks, lunch, afternoon snacks, and dinner typically last. Subsequently, global meal duration was measured, and the participants were categorized into tertiles. Obesity-related indicators, including body mass index (BMI) z score, waist circumference (WC), and skinfold thickness, were assessed. The analyses were adjusted for potential confounders such as sex, age, socioeconomic status, physical activity, sedentary behavior, diet quality, and energy intake. Results: Concerning meal duration status, adolescents with long meal durations had the lowest estimated marginal means of BMI z score, WC, and body fat percentage (using the sum of triceps and calf skinfolds). However, significant differences between adolescents with a long meal duration and those with a short meal duration were observed only for BMI z score (*p* = 0.008), and WC (*p* = 0.020). Furthermore, significant differences in BMI z score (*p* = 0.017) between adolescents with a long meal duration and those with a moderate meal duration were identified. Conclusions: These findings underscore the importance of promoting slower eating habits as part of obesity prevention strategies. Future studies should explore the causality of this association and its potential for behavioral interventions.

## 1. Introduction

Childhood and adolescent obesity are rapidly emerging as critical public health issues worldwide [1,2,3]. In recent decades, the prevalence of childhood obesity has increased alarmingly across various countries. For instance, in the United States, the prevalence of obesity among children and adolescents has more than tripled since the 1970s [4]. Similarly, countries such as the United Kingdom, Australia, and China have also reported significant increases in childhood obesity rates [5]. This rise affects not only the physical health of children and adolescents but also their psychological and social development [1,3,6,7]. Additionally, childhood obesity increases the risk for adult obesity and associated comorbidities such as type 2 diabetes, cardiovascular disease, and cancer. The growing prevalence of this epidemic can be attributed to its multifactorial nature involving genetic, behavioral, and environmental factors that tip the balance between energy intake and expenditure [2,8].

Adolescence is a stage of development characterized by profound physiological and psychosocial changes [9]. During this period, adolescents experience accelerated growth, which leads to a significant increase in nutritional and energy requirements [10]. At the same time, the hormonal changes characteristic of adolescence alter the limbic system, influencing emotional responses related to pleasure and reward seeking [9]. Additionally, this transitional phase of growth is marked by the development of self-perception and body image, which are strongly influenced by external factors such as family, peers, and social media [11,12,13,14,15,16,17]. These physiological, hormonal, emotional, and societal changes often directly impact eating behaviors, putting adolescents at risk of developing eating disorders and obesity [18,19].

The duration of meals refers to the amount of time spent eating during a single meal [20]. This behavior is often altered in adolescence and can contribute to several pathologies [21]. Due to the fast-paced nature of modern life, adolescents frequently eat hurried meals, driven by tight schedules for school, extracurricular activities, and family commitments [13,18,19,22,23,24]. In contrast, the daily eating window, as studied by Townley et al. [17]. in their systematic review, refers to the total duration of time in a day during which food is consumed. Children and adolescents have an average eating window of 11.3 h per day (95% confidence interval [CI] 11.0 to 11.7). Understanding the distinction between these two concepts is critical as both meal duration and the daily eating window could independently influence obesity-related outcomes [25]. The duration of individual meals affects the acute regulation of hunger and satiety [26], while the daily eating window plays a significant role in long-term metabolic regulation and energy balance [17]. Although there is no direct association between the speed of eating, the number of meals eaten per day, and the development of obesity, studies suggest that adolescents often skip meals and compensate with highly calorically dense foods later in the day [27,28,29,30]. Over time, these behaviors can contribute to the development of obesity, setting the stage for a range of associated health problems in both the short and long term [31].

Studies have shown that consuming meals slowly allows for a more gradual and controlled movement of food in the gastrointestinal tract [32,33,34]. Appetite and satiety, which directly affect overall body weight, are controlled by the interplay of long-acting hormones such as leptin and insulin with short-acting gastrointestinal peptides such as ghrelin, peptide YY (PYY), glucagon-like peptide (GLP-1), and cholecystokinin (CCK) [32,35]. These signals are secreted at determined times throughout the day and are directly influenced by feeding behaviors that alter the timing and speed of meals [32,34,36,37,38]. A study comparing the effects of eating speed on the release of appetite/satiety signals showed that the plasma levels of the satiety hormones GLP-1 and PYY increased more significantly in individuals who consumed a 675 kcal meal (i.e., ice cream) in 30 min than in those who ate the same meal in 5 min [39]. Similarly, a crossover study showed that decreasing the speed of eating in both lean and individuals with obesity resulted in higher postprandial serum concentrations of GLP-1 and CCK [36]. This effect was also observed in adolescents with overweight, where longer eating times led to the increased secretion of PYY and the inhibition of the hunger hormone ghrelin postprandially [37]. Consequently, a shorter mealtime duration may directly impact the perception of the amount of food required for daily needs, leading to inadequate portions and excess energy intake [38].

Eating speed not only influences appetite/satiety signals but is also associated with a greater risk of obesity and its comorbidities. For instance, a cross-sectional study of 7972 adults aged 18–65 years from Beijing (China) revealed that those who reported very fast eating behaviors had a 59% greater probability of having hypertriglyceridemia than those who reported eating slowly [39]. Similarly, other studies have corroborated these findings, showing that fast eaters tend to have greater waist–hip circumference and more obesity-associated comorbidities, such as insulin resistance, higher lipid profiles, higher triglyceride levels, higher blood pressure, and higher cholesterol levels, than those who reported slower eating speeds [39,40,41,42,43,44,45]. Childhood eating behaviors, including eating speed, have been shown to impact susceptibility to developing adult obesity. Improving these behaviors through actionable changes significantly decreases this probability [22,23,24,40]. In line with this, a three-year randomized controlled trial in 106 children and adolescents aged 9–17 years introduced a weight loss intervention with the use of a mandometer. Both adolescents and their parents were trained on how to use this device, which helps normalize eating speed to match the user’s satiety. This intervention resulted in significant weight loss one year after implementation, which was maintained at the follow-up one year later [46]. In addition, behavioral approaches such as time-restricted feeding and increasing chewing rates have long been validated in the adult population. Several studies have suggested that increasing meal duration is related to lower body weight and obesity-related indicators [47,48,49,50]. Consequently, these strategies can potentially be used as effective obesity prevention methods in children and adolescents.

To effectively address the growing challenge of childhood and adolescent obesity, comprehensive strategies must be implemented, focusing on both behavioral and environmental factors. Leveraging behavioral approaches such as time-restricted feeding and increasing the chewing rate, which have proven effective in adults, offers promising alternatives for preventing obesity in younger populations. By integrating these strategies into public health initiatives, we can help mitigate the rising prevalence of obesity and its associated health risks in future generations. However, there remains an unmet need for tailored interventions that specifically address the impact of unique eating behaviors on obesity in adolescents. Thus, in this study, we aimed to investigate the association between meal duration and obesity-related factors in a sample of Spanish adolescents aged between 12 and 17 years.

## 2. Materials and Methods

### 2.1. Study Design and Population

The current cross-sectional study constitutes a secondary analysis of data collected from the Eating Healthy and Daily Life Activities (EHDLA) project, with its protocol previously published [51]. The adolescents who participated in this research were Spanish students aged between 12 and 17 years (median = 14.0; interquartile range [IQR] = 2.0) who were attending three secondary schools in the *Valle de Ricote* (Region of Murcia, Spain). The data were collected during the 2021–2022 academic year. From the initial cohort of 1378 adolescents (100.0%) from the EHDLA study, 119 (8.7%) were excluded due to a lack of anthropometric information. Additionally, participants were excluded for missing data on overall meal duration (*n* = 464; 33.7%), overall sleep duration (*n* = 33; 2.4%), or physical activity (*n* = 7; 0.5%). Overall, 755 adolescents (45.2% boys) were included in this cross-sectional study. Written consent was obtained from the parents or guardians of the adolescent participants in this study. The participants were provided with comprehensive information regarding the study’s goals, as well as the assessments and surveys that would be conducted. The adolescents themselves were also asked to provide their consent to participate.

This research was authorized by the Bioethics Committee of the University of Murcia and the Ethics Committee of the Albacete University Hospital Complex, in addition to the Albacete Integrated Care Management (approval ID: 2218/2018 and 2021-85, respectively). The study followed the ethical principles set forth in the Helsinki Declaration.

### 2.2. Procedures

#### 2.2.1. Meal Duration (Independent Variable)

To evaluate overall meal duration, participants were asked how long (on average) breakfast, morning snacks, lunch, afternoon snacks, and dinner typically last. The possible response times were as follows: (a) I do not eat this meal (i.e., 0 min); (b) I eat it in 5 min; (c) I eat it in 10 min; (d) I eat it in 15 min; (e) I eat it in 30 min; (f) I eat it in 45 min; (g) I eat it in 60 min; or (h) I eat it in more than 60 min. The duration of all meals was summed to obtain the overall time spent on meals. Responses of more than one hour were considered a duration of 60 min. Additionally, given the absence of specific cutoff points and with the aim of obtaining balanced groups of participants, the overall duration of meals was divided into quartiles as follows: “short meal duration”, “moderate meal duration”, or “long meal duration”. As a sensitivity analysis, the same procedure was performed including only the main meals (i.e., lunch, and dinner).

#### 2.2.2. Obesity-Related Indicators (Dependent Variables)

The body weight and height of the adolescents were measured with high accuracy using an electronic scale (Tanita BC-545, Tokyo, Japan) and a portable height rod (Leicester Tanita HR 001, Tokyo, Japan), respectively. Body mass index (BMI) was calculated by dividing the body weight in kilograms by the square of the height in meters. Additionally, the BMI z score was determined using age-specific and sex-specific thresholds provided by the World Health Organization (WHO) [52]. The BMI z score indicates how many standard deviations a child’s BMI is from the mean BMI of a reference population (i.e., according to the WHO criteria [52] in this case. Waist circumference was measured to the nearest 0.1 cm at the level of the umbilicus using constant tension tape. Skinfold measurements were taken at the triceps and medial calf using calibrated steel calipers (Holtain, Crosswell, Crymych, UK) with an accuracy to the nearest 0.2 mm. These measurements have been proven to be more effective in determining body fat percentage than body mass index [53,54]. The sum of both skinfolds was computed, and the body fat percentage was estimated using the equation method of Slaughter et al. [54].

#### 2.2.3. Covariates

Adolescents voluntarily supplied information about their age and sex. The researchers used the Family Affluence Scale (FAS-III) [55] to evaluate the socioeconomic status of the participants. This assessment involved adding up the responses to six items concerning the family’s possessions, including the number of bedrooms, vehicles, bathrooms, computers, vacations, and dishwashers. The FAS-III score ranged from 0 to 13, with higher scores indicating a more privileged socioeconomic status. The researchers utilized the Mediterranean Diet Quality Index in Children and Adolescents (KIDMED) [56] to gauge adherence to the Mediterranean Diet. They used a self-administered food frequency questionnaire that had been previously validated for use in the Spanish population to assess participants’ energy intake [57]. The participants reported their typical bedtime and wake-up time on both weekdays and weekends, and the researchers calculated the average sleep duration during weekdays and weekends using the formula [(average sleep duration on weekdays × 5) + (average sleep duration on weekends × 2)] divided by 7. To assess physical activity and sedentary behavior, the researchers employed the Youth Activity Profile Physical (YAP) questionnaire [58]. This self-administered questionnaire covered a 7-day period and included 15 different items categorized into sections such as out-of-school activities, school-related activities, and sedentary habits.

### 2.3. Statistical Analysis

Methods such as density and quantile–quantile plots were used to evaluate the normal distribution of the variables, supplemented by the Shapiro–Wilk test. Categorical variables are presented as counts (*n*) and percentages (%), while continuous variables are presented as medians and IQRs. The Kruskal–Wallis *H* test was used to verify significant differences among groups. Subsequently, post hoc tests were used to determine significant differences between groups through the Wilcoxon rank sum test with continuity correction. Given that there was no interaction between overall meal duration and sex in relation to any obesity-related indicator (*p* > 0.05 for all), both girls and boys were examined jointly. To verify the relationships of meal duration (in minutes) or status (i.e., “short meal duration”, “moderate meal duration”, or “long meal duration”) with obesity-related indicators in adolescents, generalized linear regression models (GLMs) were generated. These models were constructed by applying robust methods due to their numerous benefits in addressing heteroscedasticity and outliers [22]. Thus, for continuous outcomes, GLMs with a Gaussian distribution with the “*SMDM*” method were applied (i.e., using an S-estimate, followed by an M-estimate, a design adaptive scale estimate, and another M-step). In addition, estimated marginal means (*M*) and their 95% confidence interval (CI) of each type of obesity-related indicator according to the meal duration were computed. All the models were adjusted for several covariates, including sex, age, socioeconomic status, overall sleep duration, physical activity, sedentary behavior, adherence to the Mediterranean diet, and energy intake. All the statistical analyses were performed using R statistical software (version 4.4.0) developed by the R Core Team in Vienna, Austria, and RStudio (2024.04.1 + 748) from Posit in Boston, MA, USA. A *p* value < 0.05 indicated statistical significance.

## 3. Results

Table 1 displays the main characteristics of the sample of adolescents assessed. Overall, the median meal durations for breakfast, morning snack, lunch, afternoon snack, and dinner (in minutes) were 10 (IQR = 10), 30 (IQR = 15), 30 (IQR = 15), 10 (IQR = 10), and 10 (IQR = 10) minutes, respectively. On the other hand, the adolescents who had the longest overall meal duration had the lowest median BMI z score (−0.3; IQR = 11.4) and WC (69.5; IQR = 11.4).

**Figure 1:**
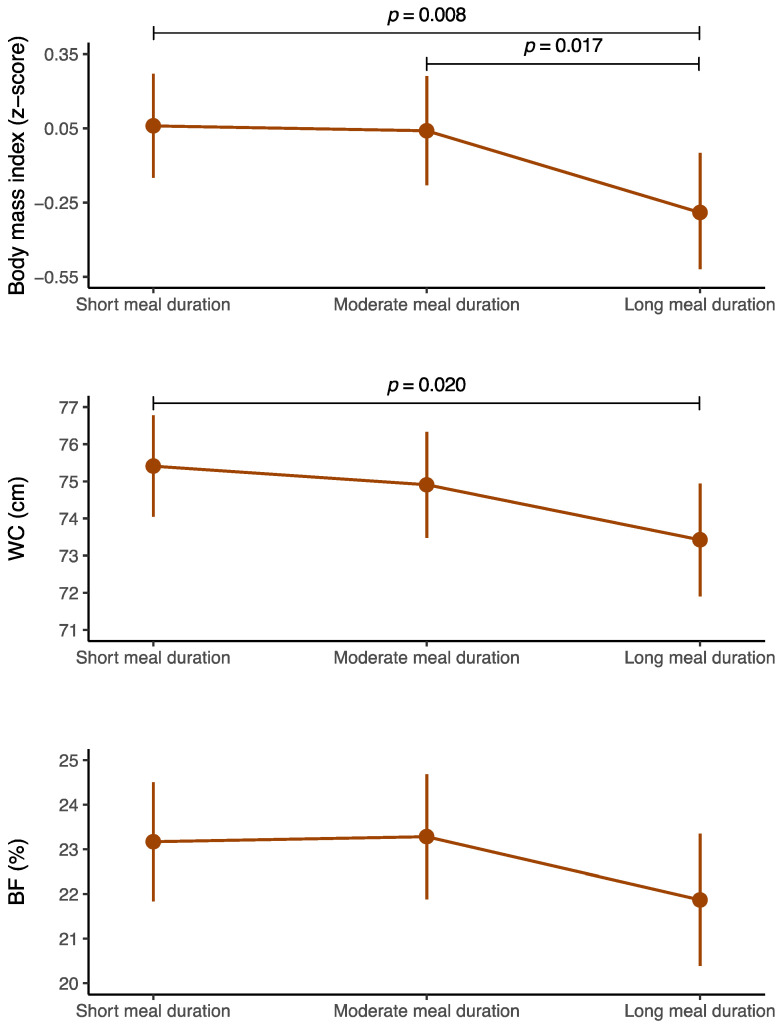
Estimated marginal means of each obesity-related indicator based on meal duration status in adolescents. The data are expressed as estimated marginal means 95% confidence intervals (vertical lines). WC, waist circumference; BF, body fat. Body mass index z score according to the World Health Organization criteria [52]. Body fat (%) according to the equation of Slaughter et al. [54].

Figure 1 displays the estimated marginal means or predictive probabilities (with their 95% CIs) of each obesity-related indicator based on meal duration status. Adolescents with long meal durations had the lowest estimated marginal means of BMI z score, WC, and body fat percentage (using triceps and calf skinfolds). However, significant differences between adolescents with long meal durations and those with short meal durations were observed only for the estimated marginal means of BMI z score (*p* = 0.008), and WC (*p* = 0.020). Furthermore, significant differences in the estimated marginal means of BMI z score (*p* = 0.017) between adolescents with long meal durations and those with moderate meal durations were identified. Conversely, there were no significant differences in the estimated marginal means of WC between adolescents with moderate meal durations and those with short meal durations (*p* = 0.551) nor between adolescents with long meal durations and those with moderate meal durations (*p* = 0.285). Additionally, there was no significant association between meal duration status and body fat percentage (*p* = 0.119). The unstandardized beta values for these associations can be found in Table 2. Moreover, the complete results of the robust GLMs examining the association between global meal duration and each obesity-related indicator are found in Tables S1–S3. Moreover, a sensitivity analysis showing the estimated marginal means of BMI z-score, WC, and body fat percentage, considering only the duration of the main meals (lunch and dinner), can be found in Tables S4–S6. Overall, these further results seem to follow the same trend.

## 4. Discussion

This study contributes to the growing body of research that underscores the association of unhealthy dietary habits with obesity-related indicators [3,6,17,22,23,59,60,61,62,63,64,65,66,67]. Our findings suggested that adolescents with longer overall meal durations tend to have a lower BMI z score and WC than their counterparts with shorter meal durations. Notably, significant differences in BMI z-scores were observed between adolescents with long meal durations compared to those with short and moderate meal durations, while significant differences in WC were observed between long and short meal durations. These associations remained significant even after adjusting for potential confounding factors such as sex, age, socioeconomic status, sleep duration, physical activity, sedentary behavior, adherence to a Mediterranean diet, and energy intake, which strengthens the robustness of the findings obtained. These results are in line with previous studies that showed that fast-eating children and adolescents were more likely to develop obesity-related indicators [40,68,69]. Despite the scarcity of causality data between the role of meal duration and obesity-related markers among young people, there are several possible reasons for our results.

There is an evident effect of meal duration on the physiology of appetite and satiety. Shorter meal durations are less likely to allow for the proper release of satiety hormones such as CCK, GLP-1, and PYY. This can in turn impact energy intake, since a lower reception of satiety signals in the hypothalamus is associated with higher food intake and lower hunger regulation [18]. Additionally, chewing food stimulates the production of saliva and gastric juices that improve the digestive process and promote adequate timing of food transit in the intestines [32,70]. This results in a regulated release of insulin that corresponds to the postprandial spike of blood glucose [33]. Fast eaters often do not chew properly on their meals, thus contributing to the incorrect release of satiety signals [68]. In addition, abrupt changes in the insulin/glucose interactions caused by a fast consumption of food are also associated with lower hunger signaling and increased energy intake [33,35].

In addition to the physiological impact of rapid eating, it is worth noting that fast eating is often linked to mindless eating (i.e., a lack of attention given to consumption due to external cues in the environment [71]). This involves consuming food without awareness of the type and quantity of food being ingested [68,72,73,74]. Mindless eaters are typically distracted by activities such as watching TV, using a computer, or talking to others [74]. Furthermore, distracted eating frequently involves convenient, on-the-go meals such as snacks that are high in calories and can trigger sharp insulin spikes, leading to poor satiety signals [50,75,76,77]. These foods are not only highly palatable but also potentially addictive, making them particularly appealing to children and adolescents [76,77]. Coupled with the increasing influence of social media and technological dependence, these factors render young people especially susceptible to the effects of mindless eating and its contribution to obesity [68,72,78]. In fact, mindfulness and aware eating behaviors have been proposed as feasible treatment interventions for young people and adults with obesity [72,73,78,79]. However, it is worth highlighting that while longer eating times are generally associated with a lower BMI and reduced obesity prevalence [40,68,69], this association is undermined when these extended eating periods are accompanied by higher food and energy intake in children. In such cases, children tend to have higher weights despite the longer meal duration [67]. These effects can also be confounded by external factors such as interactions with others, the time of day, and the type of meal consumed (e.g., breakfast, lunch). In this context, it has been pointed out that social facilitation, which predominantly impacts meal size during breakfast, can potentially override the effects of eating speed [80].

It is important to note some limitations of this study. For instance, the design of the study is cross-sectional, which limits the ability to establish causality. In addition, further research, such as longitudinal studies, is needed to establish the direction of this association. Additionally, the self-reported nature of the data for meal duration and other lifestyle factors may introduce measurement bias. Furthermore, these results are limited by the categorical nature of the meal duration data, which were reported at broad intervals rather than at precise times, potentially leading to inaccuracies when summing the total duration of all meals. Consequently, the aggregated meal duration values may not accurately reflect the true time spent on meals. Moreover, this study may also have limited application to broader populations because, although the EHDLA study included a representative sample to determine the prevalence of overweight/obesity, this study is a secondary analysis that includes only the complete information of the participants examined, so representativeness cannot be guaranteed. In addition, this study was conducted in adolescents from the Region of Murcia (Spain), the region where the prevalence of overweight/obesity is the highest in Spain [81,82]. Therefore, the results may not be generalizable to the entire Spanish population. In addition, obesity is a highly complex and multifactorial disease with diverse contributing factors [8]. Despite our efforts to adjust for numerous covariates, these determinants may still have impacted our findings. Although information was requested on the duration of meals, the reported length does not correspond only to the time spent chewing, nor does it account for possible distractors such as conversations with others or screening (i.e., watching TV, using the mobile, etc.). It would be interesting to know in future studies whether more or less chewing time is associated with more desirable obesity-related indicators. Despite these limitations, this study has several notable strengths. Specifically, our results align with trends observed in children and adolescents from various geographical locations, demonstrating the potential widespread impact of our work. Moreover, our methodology includes robust statistical techniques and adjustments for potential confounders to ensure the reliability of our findings.

## 5. Conclusions

Our findings highlight the importance of longer meal duration as a modifiable behavior in the prevention of obesity. More specifically, our results suggested that a longer meal duration is related to a lower BMI (z score) and WC. Furthermore, adolescents with a global meal duration of greater than 95 min seemed to have a lower BMI z score and WC than their counterparts with shorter meal durations. Based on the physiological effects of meal timing on satiety as well as the observational results that suggest that there is also a strong association with body weight, targeting this behavior could serve as an important and feasible tool for assessing weight loss in children and adolescents. These findings highlight the critical importance of collaborative efforts by parents and educators to improve eating behaviors related to meal duration among adolescents.

## Figures and Tables

**Table 1:** Descriptive data of the study participants according to overall meal duration status (*N* = 755).

Variable		Meal Duration Status		
		Short (≤70 min)	Moderate (71 to 95 min)	Long (≥96 min)
Participants	*n* (%)	287 (38.0)	235 (31.1)	233 (30.9)
Age (years)	Median (IQR)	14.0 (2.0)	14.0 (2.0)	14.0 (2.0)
Sex	Boys (%)	130 (45.3)	120 (51.1)	91 (39.1)
	Girls (%)	157 (54.7)	115 (48.9)	142 (60.9)
FAS-III (score)	Median (IQR)	8.0 (2.0)	8.0 (2.0)	**9.0 (3.0) ^b^**
YAP-S physical activity (score)	Median (IQR)	2.6 (0.9)	2.6 (0.9)	**2.7 (1.0) ^a^**
YAP-S sedentary behaviors (score)	Median (IQR)	2.6 (0.8)	2.6 (0.8)	**2.4 (0.6) ^a,b^**
Overall sleep duration (minutes)	Median (IQR)	497.1 (75.0)	497.1 (62.1)	**497.1 (68.6)**
KIDMED (score)	Median (IQR)	7.0 (3.0)	7.0 (3.0)	**7.0 (4.0) ^a,b^**
Energy intake (kcal)	Median (IQR)	2523.9 (1485.8)	2575.1 (1344.6)	2712.6 (1544.8)
Breakfast duration (minutes)	Median (IQR)	5.0 (5.0)	**10.0 (10.0) ^a^**	**15.0 (5.0) ^a,b^**
Lunch duration (minutes)	Median (IQR)	15.0 (0.0)	**30.0 (0.0) ^a^**	**45.0 (15.0) ^a,b^**
Dinner duration (minutes)	Median (IQR)	15.0 (5.0)	**30.0 (0.0) ^a^**	**45.0 (15.0) ^a,b^**
Morning snack duration (minutes)	Median (IQR)	10.0 (5.0)	**10.0 (5.0) ^a^**	**15.0 (20.0) ^a,b^**
Afternoon snack duration (minutes)	Median (IQR)	5.0 (5.0)	**10.0 (5.0) ^a^**	**15.0 (5.0) ^a,b^**
Global meal duration (minutes)	Median (IQR)	55.0 (15.0)	**85.0 (10.0) ^a^**	**120.0 (35.0) ^a,b^**
BMI (z score) ^†^	Median (IQR)	0.0 (1.7)	0.1 (2.2)	**–0.3 (2.0) ^a,b^**
WC (cm)	Median (IQR)	71.6 (13.2)	71.0 (14.0)	**69.5 (11.4) ^a,b^**
Skinfold triceps (mm)	Median (IQR)	15.0 (9.0)	15.0 (10.0)	14.5 (10.0)
Skinfold calf (mm)	Median (IQR)	15.0 (9.2)	15.5 (11.0)	15.0 (10.0)
Body fat (%) ^‡^	Median (IQR)	23.4 (11.6)	24.6 (12.8)	22.8 (11.8)

BMI, body mass index; FAS-III; Family Affluence Scale-III; IQR, interquartile range; KIDMED, Mediterranean Diet Quality Index for Children and Adolescents; WC, waist circumference; YAP-S, Spanish Youth Active Profile. ^†^ According to the World Health Organization criteria [28]. ^‡^ According to the equation of Slaughter et al. [54]. ^a^ Significant difference for short meal duration status (*p* < 0.05). ^b^ Significant difference for moderate meal duration status (*p* < 0.05). Values in bold indicate those where statistical significance was achieved (*p* < 0.05).

**Table 2:** Associations between meal duration and obesity-related indicators among adolescents.

Predictor		*B*	SE	LLCI	ULCI	*p* Value
BMI (z score) ^†^	Short meal duration (≤70 min)	Reference				
	Moderate meal duration (71 to 95 min)	–0.021	0.129	–0.274	0.232	0.872
	Long meal duration (≥96 min)	–0.347	0.131	–0.604	–0.090	0.008
WC (cm)	Short meal duration (≤70 min)	Reference				
	Moderate meal duration (71 to 95 min)	–0.504	0.845	–2.164	1.155	0.551
	Long meal duration (≥96 min)	–1.987	0.854	–3.664	–0.310	0.020
Body fat (%) ^‡^	Short meal duration (≤70 min)	Reference				
	Moderate meal duration (71 to 95 min)	0.114	0.825	–1.505	1.733	0.890
	Long meal duration (≥96 min)	–1.300	0.834	–2.937	0.336	0.119

The data are shown as unstandardized beta coefficients, standard errors, 95% lower and upper limit confidence intervals, and *p* values. Adjusted for sex, age, socioeconomic status, overall sleep duration, physical activity, sedentary behavior, adherence to the Mediterranean diet, and energy intake. BMI, body mass index; WC, waist circumference; *B*, unstandardized beta coefficient; SE, standard error; LLCI, lower limit confidence interval; ULCI, upper limit confidence interval. ^†^ According to the World Health Organization criteria [52]. ^‡^ According to the equation of Slaughter et al. [54].

## Data Availability

The data used in this study are available upon request from the corresponding authors. However, given that the participants are minors, privacy and confidentiality must be respected.

## Supplementary Materials

The following supporting information can be downloaded at: https://www.mdpi.com/article/10.3390/nu16162769/s1. Table S1: Full results of the robust generalized linear model evaluating the association between meal duration status and body mass index (z-score) among adolescents; Table S2: Full results of the robust generalized linear model evaluating the association between meal duration status and waist circumference among adolescents; Table S3: Full results of the robust generalized linear model evaluating the association between meal duration status and body fat (percentage) among adolescents; Table S4: Full results of the robust generalized linear model evaluating the association between meal duration status (considering only lunch and dinner) and body mass index z score among adolescents; Table S5: Full results of the robust generalized linear model evaluating the association between meal duration status (considering only lunch and dinner) and waist circumference among adolescents; Table S6: Full results of the robust generalized linear model evaluating the association between meal duration status (considering only lunch and dinner) and body fat percentage among adolescents.
