# Supplementary material for: Meal Duration and Obesity-Related Indicators among Adolescents: Insights from the EHDLA Study
Source: Nutrients. 2024 Aug 20;16(16):2769. doi: 10.3390/nu16162769 (PMC11356952; doi:10.3390/nu16162769)
Supplement: Supplementary file 1 [file nutrients-16-02769-s001.zip › nutrients-3131156-supplementary.pdf]

Supplementary material

Table S1. Full results of the robust generalized linear model evaluating the association between meal duration status and body mass index (z-score) among adolescents.

Predictor	*B*	SE	LLCI	ULCI	*p*-value
Short meal duration	Reference				
Moderate meal duration	–0.02	0.13	–0.27	0.23	0.872
Long meal duration	–0.35	0.13	–0.60	–0.09	0.008
Age (per one year)	0.07	0.04	–0.0003	0.14	0.051
Boys					
Girls	0.10	0.11	–0.11	0.32	0.351
FAS-III (per one point)	–0.04	0.03	–0.09	0.01	0.164
YAP-S physical activity (per one point)	–0.02	0.08	–0.19	0.14	0.776
YAP-S sedentary behaviors (per one point)	–0.04	0.07	–0.16	0.09	0.576
Overall sleep duration (per one hour)	0.18	0.10	–0.01	0.36	0.063
KIDMED (per one point)	0.04	0.02	–0.01	0.08	0.130
Energy intake (per 1000 kcals)	–0.03	0.03	–0.08	0.02	0.287

B, unstandardized beta coefficient; FAS-III, Family Affluence Scale-III; YAP-S, Spanish Youth Active Profile. KIDMED, Mediterranean Diet Quality Index in children and adolescents; LLCI, lower limit confidence interval; SE, standard error; ULCI, upper limit confidence interval.

Table S2. Full results of the robust generalized linear model evaluating the association between meal duration status and waist circumference among adolescents.

Predictor	*B*	SE	LLCI	ULCI	*p*-value
Short meal duration	Reference				
Moderate meal duration	–0.19	0.95	–2.05	1.68	0.846
Long meal duration	–2.10	0.90	–3.86	–0.33	0.020
Age (per one year)	0.63	0.24	0.17	1.10	0.008
Boys	Reference				
Girls	–4.54	0.73	–5.97	–3.11	0.000
FAS-III (per one point)	–0.10	0.17	–0.43	0.24	0.570
YAP- S physical activity (per one point)	0.20	0.55	–0.87	1.28	0.713
YAP-S sedentary behaviors (per one point)	–0.58	0.43	–1.42	0.27	0.181
Overall sleep duration (per one hour)	1.01	0.62	–0.21	2.24	0.104
KIDMED (per one point)	0.29	0.16	–0.02	0.60	0.066
Energy intake (per 1000 kcals)	–0.10	0.18	–0.44	0.25	0.582

B, unstandardized beta coefficient; FAS-III, Family Affluence Scale-III; YAP-S, Spanish Youth Active Profile. KIDMED, Mediterranean Diet Quality Index in children and adolescents; LLCI, lower limit confidence interval; SE, standard error; ULCI, upper limit confidence interval.

Table S3. Full results of the robust generalized linear model evaluating the association between meal duration status and body fat (percentage) among adolescents.

Predictor	*B*	SE	LLCI	ULCI	*p*-value
Short meal duration	Reference				
Moderate meal duration	1.11	0.93	–0.71	2.93	0.234
Long meal duration	–0.59	0.88	–2.32	1.13	0.502
Age (per one year)	–0.64	0.23	–1.10	–0.18	0.006
Boys	Reference				
Girls	2.52	0.71	1.12	3.92	<0.001
FAS-III (per one point)	–0.05	0.17	–0.38	0.27	0.742
YAP-S physical activity (per one point)	–0.44	0.54	–1.49	0.61	0.413
YAP-S sedentary behaviors (per one point)	0.29	0.42	–0.53	1.12	0.487
Overall sleep duration (per one hour)	1.01	0.61	–0.19	2.20	0.100
KIDMED (per one point)	0.13	0.15	–0.17	0.43	0.398
Energy intake (per 1000 kcals)	–0.22	0.17	–0.56	0.12	0.208

B, unstandardized beta coefficient; FAS-III, Family Affluence Scale-III; YAP-S, Spanish Youth Active Profile. KIDMED, Mediterranean Diet Quality Index in children and adolescents; LLCI, lower limit confidence interval; SE, standard error; ULCI, upper limit confidence interval.

Table S4. Full results of the robust generalized linear model evaluating the association between meal duration status (considering only lunch and dinner) and body mass index z score among adolescents.

Predictor	*B*	SE	LLCI	ULCI	*p*-value
Short meal duration	Reference				
Moderate meal duration	–0.03	0.18	–0.39	0.34	0.886
Long meal duration	–0.21	0.18	–0.56	0.14	0.241
Age (per one year)	0.07	0.04	0.00	0.14	0.059
Boys	Reference				
Girls	0.08	0.11	–0.14	0.30	0.457
FAS-III (per one point)	–0.04	0.03	–0.09	0.01	0.153
YAP-S physical activity (per one point)	–0.03	0.08	–0.20	0.13	0.702
YAP-S sedentary behaviors (per one point)	0.18	0.10	0.00	0.37	0.054
Overall sleep duration (per one hour)	–0.04	0.07	–0.17	0.09	0.543
KIDMED (per one point)	0.03	0.02	–0.01	0.08	0.145
Energy intake (per 1000 kcals)	–0.03	0.03	–0.09	0.02	0.221

B, unstandardized beta coefficient; FAS-III, Family Affluence Scale-III; YAP-S, Spanish Youth Active Profile. KIDMED, Mediterranean Diet Quality Index in children and adolescents; LLCI, lower limit confidence interval; SE, standard error; ULCI, upper limit confidence interval.

Table S5. Full results of the robust generalized linear model evaluating the association between meal duration status (considering only lunch and dinner) and waist circumference among adolescents.

Predictor	*B*	SE	LLCI	ULCI	*p*-value
Short meal duration	Reference				
Moderate meal duration	–0.63	1.21	–3.01	1.74	0.601
Long meal duration	–1.71	1.18	–4.01	0.60	0.148
Age (per one year)	0.60	0.24	0.13	1.08	0.012
Boys	Reference				
Girls	–4.59	0.73	–6.02	–3.15	<0.001
FAS-III (per one point)	–0.12	0.17	–0.45	0.22	0.492
YAP-S physical activity (per one point)	0.16	0.55	–0.92	1.24	0.769
YAP-S sedentary behaviors (per one point)	1.08	0.63	–0.15	2.30	0.086
Overall sleep duration (per one hour)	–0.61	0.43	–1.46	0.24	0.158
KIDMED (per one point)	0.28	0.16	–0.03	0.58	0.081
Energy intake (per 1000 kcals)	–0.10	0.18	–0.45	0.24	0.559

B, unstandardized beta coefficient; FAS-III, Family Affluence Scale-III; YAP-S, Spanish Youth Active Profile. KIDMED, Mediterranean Diet Quality Index in children and adolescents; LLCI, lower limit confidence interval; SE, standard error; ULCI, upper limit confidence interval.

Table S6. Full results of the robust generalized linear model evaluating the association between meal duration status (considering only lunch and dinner) and body fat percentage among adolescents.

Predictor	*B*	SE	LLCI	ULCI	*p*-value
Short meal duration	Reference				
Moderate meal duration	0.31	1.17	–1.99	2.61	0.791
Long meal duration	–0.07	1.14	–2.30	2.17	0.954
Age (per one year)	–0.65	0.24	–1.11	–0.19	0.006
Boys	Reference				
Girls	2.49	0.72	1.09	3.90	0.001
FAS-III (per one point)	–0.08	0.17	–0.41	0.24	0.625
YAP-S physical activity (per one point)	–0.45	0.54	–1.50	0.61	0.405
YAP-S sedentary behaviors (per one point)	1.06	0.61	–0.14	2.26	0.084
Overall sleep duration (per one hour)	0.28	0.42	–0.55	1.12	0.502
KIDMED (per one point)	0.11	0.15	–0.20	0.41	0.493
Energy intake (per 1000 kcals)	–0.20	0.17	–0.54	0.14	0.257

B, unstandardized beta coefficient; FAS-III, Family Affluence Scale-III; YAP-S, Spanish Youth Active Profile. KIDMED, Mediterranean Diet Quality Index in children and adolescents; LLCI, lower limit confidence interval; SE, standard error; ULCI, upper limit confidence interval.
